# Temperature sensitivity of carbon concentrating mechanisms in the diatom *Phaeodactylum tricornutum*

**DOI:** 10.1007/s11120-023-01004-2

**Published:** 2023-03-07

**Authors:** Meng Li, Jodi N. Young

**Affiliations:** School of Oceanography, University of Washington, Seattle, WA USA

**Keywords:** Diatom, CCM, Temperature, Modeling, RuBisCO, *Phaeodactylum tricornutum*

## Abstract

Marine diatoms are key primary producers across diverse habitats in the global ocean. Diatoms rely on a biophysical carbon concentrating mechanism (CCM) to supply high concentrations of CO_2_ around their carboxylating enzyme, RuBisCO. The necessity and energetic cost of the CCM are likely to be highly sensitive to temperature, as temperature impacts CO_2_ concentration, diffusivity, and the kinetics of CCM components. Here, we used membrane inlet mass spectrometry (MIMS) and modeling to capture temperature regulation of the CCM in the diatom *Phaeodactylum tricornutum (Pt)*. We found that enhanced carbon fixation rates by *Pt* at elevated temperatures were accompanied by increased CCM activity capable of maintaining RuBisCO close to CO_2_ saturation but that the mechanism varied. At 10 and 18 °C, diffusion of CO_2_ into the cell, driven by *Pt*’s ‘chloroplast pump’ was the major inorganic carbon source. However, at 18 °C, upregulation of the chloroplast pump enhanced (while retaining the proportion of) both diffusive CO_2_ and active HCO_3_^−^ uptake into the cytosol, and significantly increased chloroplast HCO_3_^−^ concentrations. In contrast, at 25 °C, compared to 18 °C, the chloroplast pump had only a slight increase in activity. While diffusive uptake of CO_2_ into the cell remained constant, active HCO_3_^−^ uptake across the cell membrane increased resulting in *Pt* depending equally on both CO_2_ and HCO_3_^−^ as inorganic carbon sources. Despite changes in the CCM, the overall rate of active carbon transport remained double that of carbon fixation across all temperatures tested. The implication of the energetic cost of the *Pt* CCM in response to increasing temperatures was discussed.

**Supplementary Information:**

The online version contains supplementary material available at 10.1007/s11120-023-01004-2.

## Introduction

Diatoms are responsible for 40% of marine carbon fixation (Field et al. 1998) and are found across all latitudes of the ocean. Increasing anthropogenic inputs of CO_2_ into the atmosphere is driving both ocean warming and an increase in the ocean’s absorption of anthropogenic CO_2_ leading to decreasing oceanic pH (ocean acidification) and changes in oceanic carbon chemistry with increasing concentrations of dissolved CO_2_ and HCO_3_^−^ (Zeebe 2012; Cheng et al. 2022). While different marine phytoplankton species have differing responses to the elevation of CO_2_ (Dutkiewicz et al. 2015), diatoms’ ability to fix carbon appears largely insensitive to ocean acidification due to their highly effective carbon concentrating mechanisms (CCMs) that can utilize the much larger pool of HCO_3_^−^ available in seawater. Most diatoms studied to date possess a biophysical CCM that uses a compartment (pyrenoid) capable of elevating CO_2_ around the CO_2_ fixing enzyme, RuBisCO (Roberts et al. 2007; Hopkinson et al. 2016). Some diatoms may also possess a biochemical CCM (e.g., a C4 pathway) but this is still open for debate (Reinfelder et al. 2000; Morel et al. 2002; Roberts et al. 2007; Clement et al. 2016) and is not the focus of this study. While pyrenoids are common in diatoms, the mechanism by which the CCM elevates CO_2_ around RuBisCO varies among species. Broadly, there appears to be two types of CCM, (1) those where inorganic carbon (C_i_) uptake is dependent on an extracellular carbonic anhydrase (eCA) and (2) those lacking an eCA and instead rely on HCO_3_^−^ transporters on the cell membrane for C_i_ uptake (Tsuji et al. 2017a).

While algal CCMs are highly effective, they are also energy intensive (Raven 2010; Raven et al. 2014). CCMs elevate pyrenoid CO_2_ concentrations (needed to nearly saturate RuBisCO) to levels that far exceed CO_2_ concentrations in seawater, creating large concentration gradients, which encourages diffusive CO_2_ efflux across highly permeable membranes. Manipulation of internal CO_2_ pools and active transport of HCO_3_^−^ across membranes requires energy input. Numerous studies have shown that increased extracellular CO_2_ or changes in extracellular or intracellular pH can reduce the energetic requirement of the CCM (Hopkinson 2014; Mangan et al. 2016), however, there has been mixed evidence in diatoms on whether this can result in enhanced carbon fixation rates (Gao et al. 2014). Optimal CCM function requires synergistically balancing different CCM components, which facilitate HCO_3_^−^ and CO_2_ influx into the cell, recycling of leaked CO_2_ and the maintenance of high CO_2_ concentrations inside the pyrenoid.

Temperature plays a large but relatively understudied role on the CCM. As temperatures increase, the solubility of CO_2_ and O_2_ in seawater is reduced, along with a decrease in RuBisCO substrate affinity and CO_2_/O_2_ specificity (Galmés et al. 2016). However, membrane permeability, rates of diffusion and biochemical reaction rates all increase with rising temperatures, until an optimum is reached (Beardall et al. 2009; Li and Young 2022). It is likely that the temperature sensitivity of these different CCM components vary, leading to imbalanced C_i_ fluxes with fluctuating temperature. Therefore, diatoms need to regulate their CCM components to maintain balanced C_i_ fluxes to support high photosynthetic efficiency. There has been extensive research on the response of C4 plants (biochemical CCM) to varying temperature (Sage and Kubien 2007) but little is known about CCM response to temperature in the marine environment. While temperature fluctuations may not be as large in marine compared to terrestrial environments, marine heatwaves can be 5–6 °C above average temperatures and are devastating for marine ecosystems (Frölicher and Laufkötter 2018). It is likely these marine heatwaves are becoming more common, particularly in coastal regions (Lima and Wethey 2012; Hughes et al. 2018).

*Phaeodactylum tricornutum* (*Pt*) is a model coastal marine diatom with a well-established CCM model at 20 °C (Hopkinson et al. 2011; Hopkinson 2014). While *Pt* is of low abundance in the open ocean, it is widely distributed globally from sub-polar to tropical environments and able to grow between 5 and 28 °C in the lab (Kudo et al. 2000; De Martino et al. 2011; Rastogi et al. 2020). Here, we acclimated the model diatom *Pt* to three different temperatures over its growth range and measured CCM activity using membrane inlet mass spectrometry (MIMS). Experimental data were fit within the *Pt* CCM model, based on *Fragilariopsis cylindrus* (*Fcyl*) CCM (Li and Young 2022) adapted from the “chloroplast pump” model (Hopkinson 2014), to explore how the *Pt* CCM functions across temperatures.

## Materials and methods

### Cell culture conditions and growth rate

*Phaeodactylum tricornutum* CCMP632 (*Pt*) cells were cultured in buffered (25 mM HEPES, pH 8.10) f/2 media with salinity at 35‰. The pH of the culture media was adjusted to 8.10 for each growth temperature, i.e., 10 °C, 18 °C and 25 °C. Light intensity was set at ~ 120 µmol m^−2^ s^−1^ with 16 h:8 h, light:dark cycle. Cell cultures were subcultured weekly and acclimated at each temperature for at least two weeks. Initial inoculum was 1 × 10^4^ cells ml^−1^ (or 1 × 10^5^ cells ml^−1^ at 10 °C) at day zero and cells were harvested during exponential growth phase, at cell densities between 5 × 10^5^ and 1 × 10^6^ cells ml^−1^ on the day of each experiment. Growth rates were calculated as described in Li and Young (2022). Beckman Coulter Z2 cell counter was used to determine cell density and size, assuming a spherical cell shape for diameter calculation. The nonspherical shape of *Pt* cells, which determines the cell surface area, does not impact the estimation of volume-based mass transfer coefficients.

### Membrane inlet mass spectrometry

Membrane Inlet Mass Spectrometry (MIMS), including gas concentration calibrations and data analyses are as described in Li and Young (2022) for *Fcyl*. Photosynthesis and C_i_ usage measurements with *Pt* cells were carried out in a similar fashion though at different temperatures (10, 18 and 25 °C) with the presence of the eCA inhibitor acetazolamide (AZ) as detailed in previous studies (Hopkinson et al. 2011; Hopkinson 2014; Li and Young 2022). Like our *Fcyl* experiments, ^18^O, ^13^C-labeled NaHCO_3_ (final concentration ~ 2 mM) was used to determine HCO_3_^−^ dehydration rate, CO_2_/HCO_3_^−^ mass transfer coefficient (f_c_, f_b_) and CA activities at different temperatures (Li and Young 2022). *Pt* cells were concentrated before loading into the MIMS chamber with final average densities of 3 ~ 4 × 10^6^ cells ml^−1^.

### Photosynthetic rate and C_i_ uptake versus DIC concentration

Additional experiments to investigate the apparent kinetic properties of *Pt* C_i_ uptake were conducted by measuring rates of O_2_ evolution under varying dissolved inorganic carbon concentrations ([DIC]). Similar to the previous study of Hopkinson (2014), stepwise increases in [DIC] were created by loading ^13^C-NaHCO_3_ into the MIMS chamber in the presence of 25 mM HEPES buffered (pH 8.1) artificial seawater. For each different [DIC], *Pt* cells were treated to a dark–light-dark cycle until quasi-steady state was reached (see Fig. S1, e.g.). The CO_2_ and O_2_ signals were used for calculating photosynthetic rate and Ci uptake according to earlier studies (Badger et al. 1994; Hopkinson et al. 2011; Li and Young 2022). The cytosolic CO_2_ concentration was estimated using following equation:

1 $$\left[ {CO_{2} } \right]_{cyt} = \left[ {CO_{2} } \right]_{bulk} - \frac{{U_{{CO_{2} }} }}{{f_{c} }}$$

where [CO_2_]_cyt_ and [CO_2_]_bulk_ are the cytosolic and bulk CO_2_ concentrations respectively; U_CO2_ is the CO_2_ uptake rate and f_c_ is the mass transfer coefficient of CO_2_. The rate of photosynthesis (O_2_ evolution) as a function of [CO_2_] was plotted and fit to the Michaelis–Menten equation.

### RuBisCO kinetics and quantification

In vitro RuBisCO kinetics at different temperatures (6, 12, 18, 25 °C) were measured according to previous studies (Sharwood et al. 2008; Young et al. 2016). Briefly, RuBisCO active sites were quantified using [^14^C] 2-CABP binding assay as described by Sharwood et al. (2008). For the ribulose-P_2_-dependent ^14^CO_2_-fixation assays, crude extracts of soluble diatom protein were incubated with 15 mM NaH^14^CO_3_ and 15 mM MgCl_2_ at the relevant temperature for 10–15 min to activate RuBisCO. This extract was added to 7 ml septum-capped scintillation vials containing reaction buffer (0.5 ml of 100 mM EPPS-NaOH, pH 8, 15 mM MgCl_2_, 0.6 mM ribulose-P_2_, 0.1 mg ml^−1^ CA) equilibrated with 21% (v/v) O_2_ in N_2_ and five differing concentrations of ^14^CO_2_ (between 10 and 115 μM). Values for the half saturation constant for CO_2_ in air (K_Cair_) and maximal carboxylase activity in air (*V*_*Cmax*_) were extrapolated from the data using the Michaelis–Menten equation as described previously (Sharwood et al. 2008; Whitney et al. 2011). The carboxylation turnover rate of RuBisCO (*k*_*catC*_) was calculated by dividing *V*_*Cmax*_ by the number of RuBisCO active sites as determined in [^14^C] 2-CABP-binding assay.

Temperature dependence of *k*_*catC*_ and K_Cair_ was described by fitting following equation as described by Arcus et al. (2016):

2 $$lnk = a \times lnT + b \times \left( \frac{1}{T} \right) + c$$

The best-fit constants (a, b, c) were used to interpolate *k*_*catC*_ and K_Cair_ values at different temperature (T).

Western Blots were used to quantify the relative abundance of RuBisCO in *Pt* cells cultured at different temperatures, with experimental details described previously (Li and Young 2022). However, Western Blots using commercial anti-RbcL antibody and standards consistently underestimated absolute RuBisCO concentrations in diatoms. Thus, for modeling purposes, average RuBisCO quantities were estimated by assuming *Pt* RuBisCO reaches 80% saturation rate during photosynthesis at 18 °C, based on previous modeling and field research work (Hopkinson 2014, Kranz et al. 2015).

### Modeling and statistical analyses

We previously adapted the “chloroplast pump” models (Hopkinson 2014; Li and Young 2022) to simulate the C_i_ fluxes of *Fcyl*. By adjusting the HCO_3_^−^ uptake behavior observed in our *Pt* data, as well as other physiochemical parameters acquired in this study, we modified the *Fcyl* CCM model to simulate *Pt* C_i_ fluxes during steady state photosynthesis at different temperatures. The model was constrained to describe observed CO_2_ concentrations, net O_2_ evolution rates and C_i_ uptake rates. The Python script with modeling parameter files can be found on GitHub (https://github.com/limengwsu/Pt_CCM_T). All descriptive statistical analyses including mean, stand deviation, tukey HSD p values, etc. were calculated using Python packages including pandas, numpy, scipy (Virtanen et al. 2020) and statsmodels (Seabold and Perktold 2010).

## Results

### Growth rates and photosynthesis with temperature

**Fig. 1:**
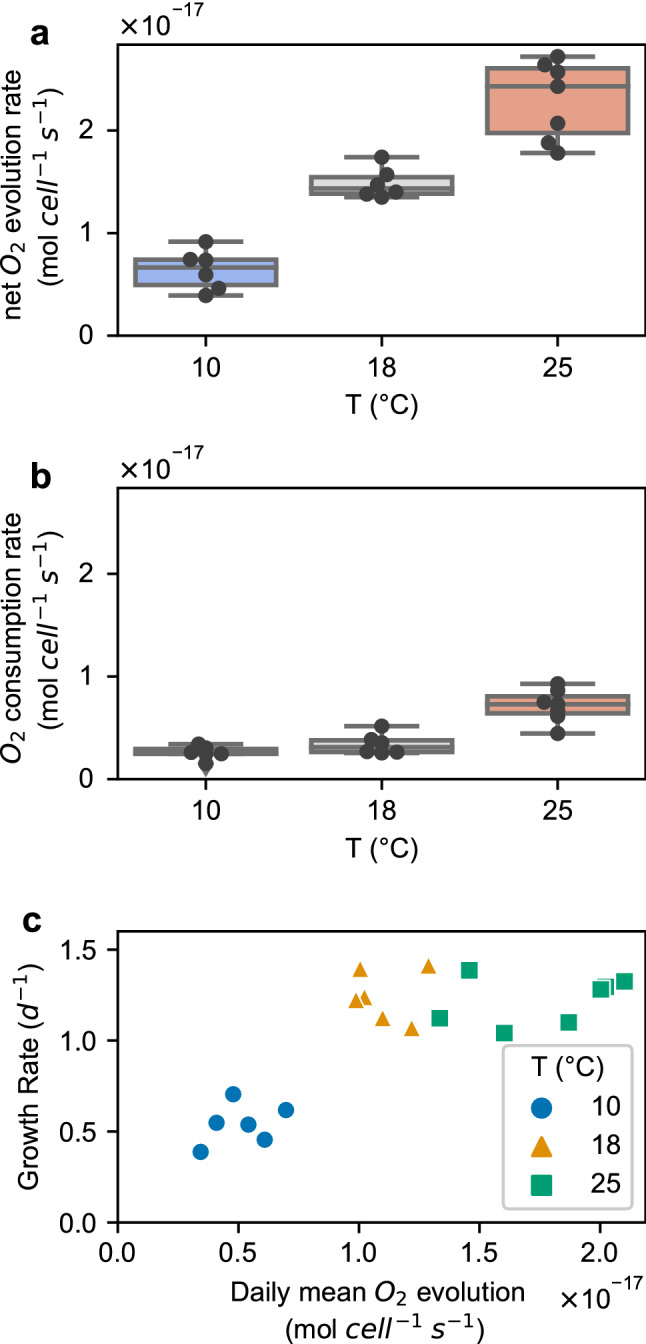
*Pt* net photosynthetic, respiratory and growth rate at different temperature. **a.** Net oxygen evolutionary rate in the light per cell across different temperatures. **b.** Oxygen consumption (dark respiratory) rate per cell across different temperatures. **c.** The correlation between growth rate and averaged daily primary production rate, presented as daily net O_2_ evolution rate per cell. All box plots have whiskers extending maximum 1.5 times of IQR from box

*Pt* net photosynthesis increased steadily across all temperatures tested, with an ~ fourfold increase between 10 and 25 °C, as measured by the rate of O_2_ evolution in the light (Fig. 1a). Respiration (O_2_ consumption in the dark) also increased significantly at 25 °C compared with 10 and 18 °C (*p* ≤ 0.001, Fig. 1b). The estimated daily net O_2_ evolution (calculated as 1/3 dark respiration and 2/3 light O_2_ evolution over a 16:8 daylight cycle), increased with temperature (*p* ≤ 0.001, Fig. 1c). However, the specific growth rate (d^−1^) of *Pt* only increased between 10 and 18 °C, not between 18 and 25 °C (Fig. 1c). This disparity could be attributed to changing rates of respiration and photosynthesis over a diel cycle that were not captured in our short-term MIMS experiments. In addition, cell size also increased significantly between 18 and 25 °C (*p* ≤ 0.001), though only by ~ 10% in volume, and may partly explain the difference observed between daily net photosynthesis and growth rates **(**Figs. 1c, S2**)**.

### RuBisCO kinetics and quantity

**Fig. 2:**
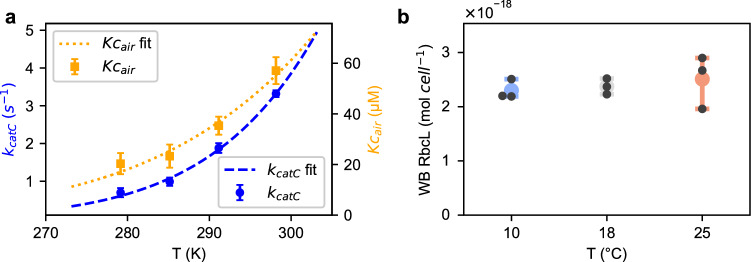
The impact of temperature on RuBisCO kinetic properties and quantity in *Pt. a.* RuBisCO carboxylation kinetic parameters *k*_*catC*_ and K_Cair_ in the presence of air. Curve fitting and interpolation were used to derived parameters at different temperatures. **b.** Western Blot (WB) quantification of RbcL in *Pt* cultured at different temperatures. Error bars represent SD with *n* = 3

Faster growth and photosynthetic rates at higher temperatures could be attributed to the temperature-driven increase in RuBisCO catalytic activity (*k*_*catC*_). From 10 to 25 °C, RuBisCO *k*_*catC*_ increased from 0.9 to 3.3 s^−1^, an almost four-fold increase between 10 and 25 °C, similar in magnitude to the increase in photosynthetic oxygen evolution (Fig. 2a). Between 10 and 25 °C, there is also a 2.5-fold increase in K_Cair_ from 22 to 55 µM (Fig. 2a), thus at higher temperatures RuBisCO needs a higher CO_2_ concentration for similar saturation levels. As K_Cair_ was less temperature sensitive than *k*_*catC*_, carboxylation efficiency (*k*_*catC*_/K_Cair_) increases with temperature.

There was little change in RuBisCO abundance per cell across temperatures in *Pt* (Figs. 2b, S3a). The Western Blot method tracks changes in relative abundance but appears to underestimate diatom RbcL quantity in *Pt*, which was also observed in our previous work on *Fcyl* (Li and Young 2022). Our estimation of average gross CO_2_ fixation rates at RuBisCO (based on estimated gross O_2_ evolution rates, Fig. S3b), increased from 0.90 to 3.0 × 10^–17^ mol cell^−1^ s^−1^ with a temperature increase from 10 to 25 °C. Dividing the gross CO_2_ fixation rates by *k*_*catC*_, the results lead to an average minimum RuBisCO quantity at 1 × 10^–17^ mol per *Pt* cell. For modeling purposes and to estimate the relative substrate (CO_2_) concentration at RuBisCO, 80% saturation was assumed at 18 °C, which resulted in an estimated abundance of 1.24 × 10^–17^ mol RuBisCO per *Pt* cell across different temperatures. Combined with K_Cair_ data, we estimated the average CO_2_ concentration around RuBisCO during steady state photosynthesis to be 95, 147, and 151 µM at 10, 18, and 25 °C, respectively.

### Inorganic carbon usage

**Fig. 3:**
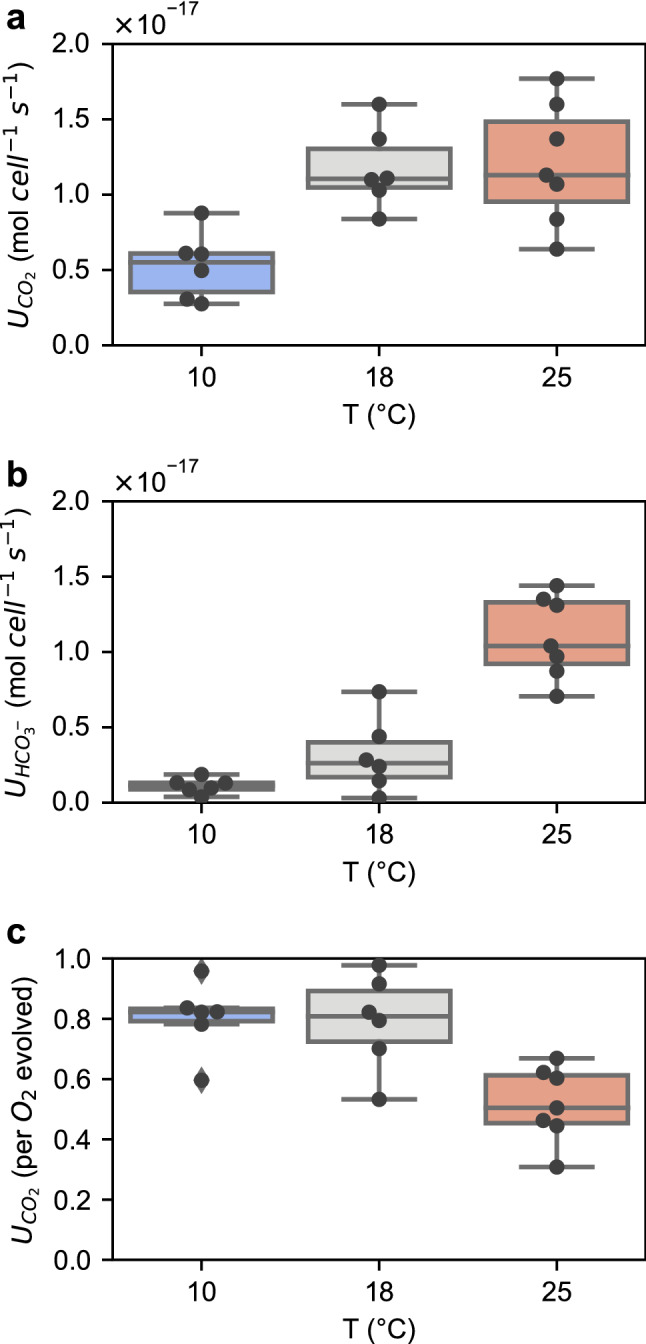
Inorganic carbon uptake by *Pt* at different temperatures*. a* and **b** Uptake rate of CO_2_ (U_CO2_) and HCO_3_^−^ (U_HCO3-_) per cell basis. **c.** Fraction of CO_2_ supply normalized to O_2_ evolution

An increase in cellular inorganic carbon (C_i_) uptake was required to supply the faster carboxylation rates and maintain higher CO_2_ concentrations within the pyrenoid with increasing temperature. Total C_i_ uptake increases at higher temperatures, which was largely driven by an increase in CO_2_ uptake from 10 to 18 °C (Fig. 3a) and by an increase in HCO_3_^−^ uptake from 18 to 25 °C (Fig. 3b). This results in the proportional supply of C_i_ in *Pt* to be ~ 80% CO_2_ at the lower temperatures of 10 and 18 °C, but only ~ 50% CO_2_ supply at 25 °C (*p* ≤ 0.006, Fig. 3c).

**Fig. 4:**
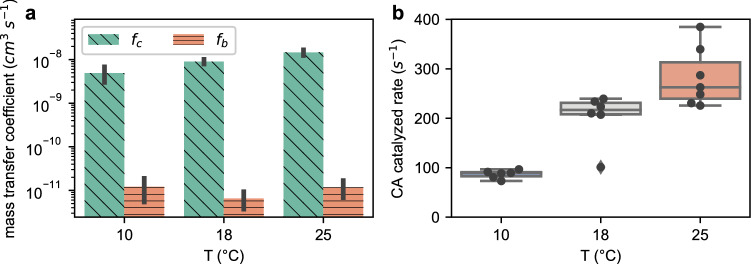
Temperature impact on *Pt* inorganic carbon mass transfer coefficient (**a)** and intracellular CA catalytic rate (**b)**

Using ^18^O-exchange method (Hopkinson et al. 2011; Li and Young 2022), we estimated the mass transfer coefficients of CO_2_ and HCO_3_^−^ (f_c_, f_b_ respectively) in *Pt* at different temperatures. The permeability of CO_2_ across the *Pt* cell membrane (and frustule), measured as f_c_, steadily increased with temperature (*p* ≤ 0.006 for each temperature pair, Fig. 4a). The permeability for HCO_3_^−^, indicated by f_b_, was three orders of magnitude lower than CO_2_ and insensitive to temperature (Fig. 4a). Thus, as noted in previous studies (Hopkinson et al. 2011), the cell membrane can be treated as essentially impermeable for HCO_3_^−^. The increased f_c_ at higher temperatures may facilitate the influx of CO_2_, where CO_2_ uptake rates are faster at higher temperatures than at 10 °C (*p* ≤ 0.007, Fig. 3b). As discussed by Hopkinson (2014), the cytosolic CA can also facilitate CO_2_ uptake. Like f_c_, the estimated catalytic rate of intracellular CA in *Pt* was higher at warmer temperatures (*p* < 0.02 for each temperature pair, Fig. 4b).

### C_i_ uptake rate versus C_i_ concentration

**Fig. 5:**
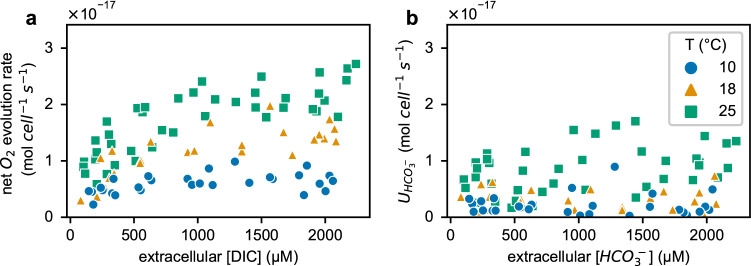
The impact of DIC (**a)** and HCO_3_^−^ (**b)** concentration on net O_2_ evolution rate and uptake rate of HCO_3_^−^ respectively. Data points were pooled from 4, 3, 5 independent cultures for temperature at 10, 18, 25 °C respectively

To understand the kinetics of C_i_ uptake by *Pt* and to model C_i_ fluxes during photosynthesis at different temperatures, we investigated the effect of [DIC] on net photosynthetic rates (Fig. 5a). As expected, lower [DIC] limited net photosynthetic rates across different temperatures. However, when C_i_ uptake was analyzed, the limitation of photosynthesis was not from the limited HCO_3_^−^ uptake under our experimental conditions. HCO_3_^−^ uptake was not responsive to bulk [HCO_3_^−^] in the range of 0.08–2 mM, regardless of temperature (Fig. 5b). This indicates that *Pt* has highly specific HCO_3_^−^ transporters that operate with half saturation constants (K_0.5_) <  < 100 µM (agreeing with earlier research on *Pt* (Nakajima et al. 2013; Tsuji et al. 2017a; Nawaly et al. 2022). The observed insensitivity of HCO_3_^−^ uptake to bulk [HCO_3_^−^] (and [DIC]) suggests an alternative entry of C_i_ into the cytosol (Fig. S4), i.e., through CO_2_ diffusion, which causes the limit on photosynthesis at low [DIC].

The diffusion of CO_2_ into the cell is driven by the CO_2_ concentration gradient between the extracellular environment and the cytosol (Hopkinson 2014). C_i_ from the cytosol is then concentrated within the chloroplast by the “chloroplast pump”. The “chloroplast pump” as defined by Hopkinson (2014) intensively “pumps” HCO_3_^−^ from the cytosol to the chloroplast, maintaining low HCO_3_^−^ concentrations within the cytosol. Rapid cytosolic-CA-mediated net flux of CO_2_ to HCO_3_^−^ then drives the diffusive influx of CO_2_ into the cytosol from outside the cell. With our measured f_c_, we estimated cytosolic CO_2_ concentrations using Eq. 1, which allowed us to investigate the relationship between cytosolic CO_2_ concentration and net photosynthetic rate (Fig. S5b). Fitting the net O_2_ evolution rate versus extracellular and cytosolic CO_2_ concentrations to the Michaelis–Menten curve separately revealed a strong “chloroplast pump”, with average K_0.5_ ≤ 1 µM for both extracellular and cytosolic CO_2_ (Fig. S5). With the equilibrium constant of CO_2_/HCO_3_^−^ interconversion being ~ 100, the K_0.5_ for HCO_3_^−^ transport from the cytosol to the chloroplast was estimated to be < 100 µM. With our experimental setup, the temperature impact on the affinity (K_0.5_) of HCO_3_^−^/CO_2_ at the chloroplast level was not distinguishable.

### Modeling the CCM with temperature

**Fig. 6:**
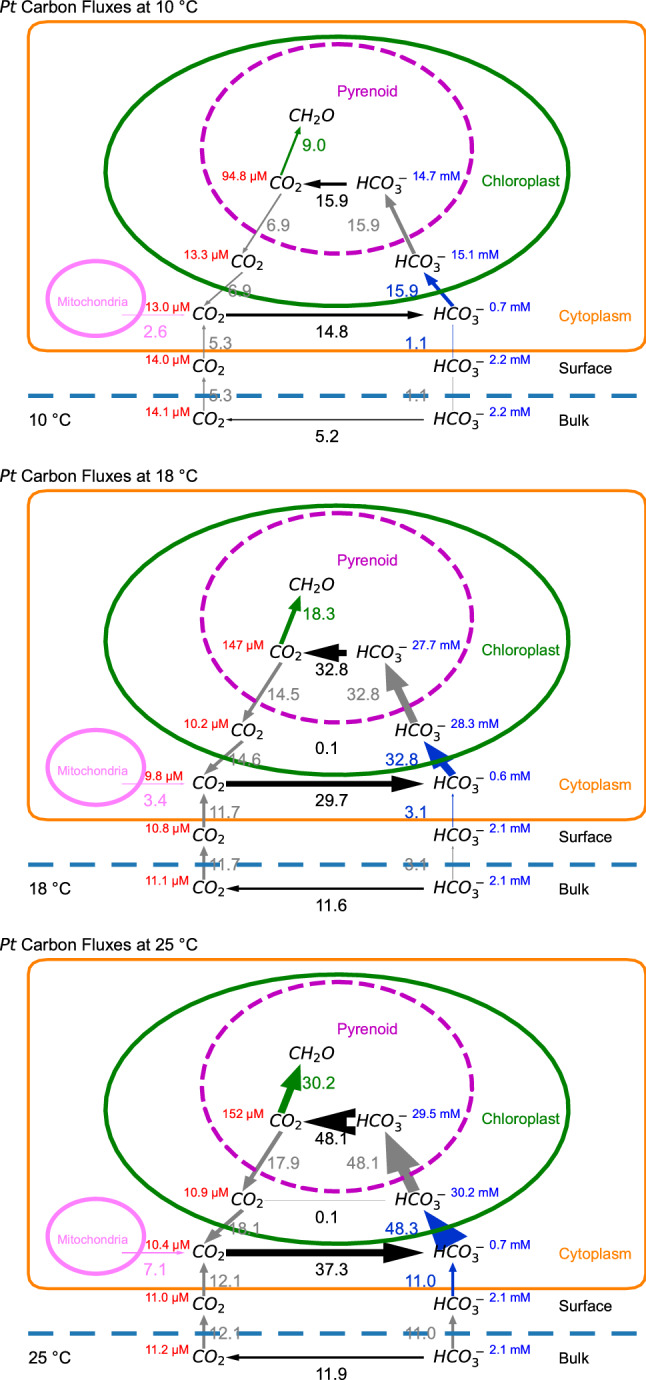
CCM models of *Pt* carbon (Ci) fluxes during photosynthesis at different temperatures. Gray, black, blue, and green arrows along with net flux rates (unit in 10^–18^ mol cell^−1^ s^−1^) represent diffusive, interconversion, active uptake, and fixation of inorganic carbon species, respectively. Arrow widths are drawn proportional to flux rates. The concentrations of CO_2_ and HCO_3_^−^ are labeled in each compartment with units. The “Surface” compartment (Hopkinson et al. 2013) denotes the cellular layer outside the cytoplasmic membrane, including periplasmic space and frustule

Combining the quantitative data of RuBisCO kinetics, CA activities, net O_2_ evolution, C_i_ uptake rates and its kinetic properties, etc., we modeled the C_i_ fluxes in *Pt* during photosynthesis at different temperatures (Fig. 6). At steady state in general, CO_2_ diffuses from the bulk environment through the surface layer and cytoplasmic membrane into the cytosol, with additional CO_2_ supplied to the cytosol from mitochondrial respiration and leakage from the chloroplast (gray arrows pointing at cytosolic CO_2_, Fig. 6). The concentrations of CO_2_ and HCO_3_^−^ in the cytosol favor the net conversion of CO_2_ to HCO_3_^−^ by cytosolic CA. The CA supplied HCO_3_^−^, combined with extracellular HCO_3_^−^ uptake through cytoplasmic membrane, is pumped into the chloroplast by potential transporter(s). This allows the accumulation of high concentrations of HCO_3_^−^ within chloroplast and subsequently in the pyrenoid, where HCO_3_^−^ is converted to CO_2_ by pyrenoid CA and fixed by RuBisCO (green arrows, Fig. 6). Due to the concentration gradient between pyrenoid and chloroplast stroma, some proportion of the CO_2_ leaks out from the pyrenoid into the chloroplast stroma and subsequently into the cytosol. As discussed by Hopkinson (Hopkinson 2014), the lack of CAs in the chloroplast stroma allows the buildup of HCO_3_^−^, with a very small fraction converted to CO_2_ before reaching the pyrenoid.

As temperature increases, the model was able to capture the experimentally observed fluxes of C_i_ uptake and photosynthetic rates, including faster diffusive CO_2_ uptake from the bulk environment to the cytosol at 18 °C compared with 10 °C, and enhanced HCO_3_^−^ uptake into the cytosol at 25 °C compared with 18 °C (Figs. 3, 6). The model also enabled visualization of the intracellular C_i_ fluxes that are less accessible through experiments. All intracellular C_i_ fluxes operate at faster rates as temperatures rise (Fig. 6). Notably the net fluxes of cytosolic CA-catalyzed conversion of CO_2_ to HCO_3_^−^, HCO_3_^−^ transport from the cytosol to the chloroplast, and the leakage of CO_2_ from the chloroplast to the cytosol are faster at higher temperatures. For every mole of HCO_3_^−^ transported into the chloroplast, there are 0.37, 0.45, and 0.43 mol of CO_2_ leaking out of the chloroplast at 10, 18, and 25 °C, respectively. In other words, for every carbon fixed by RuBisCO, there are 1.6, 1.8 and 1.8 HCO_3_^−^ molecules transported into the chloroplast at 10, 18, and 25 °C, respectively. When HCO_3_^−^ uptake from the surface/bulk environment into the cytosol is included (i.e., total active HCO_3_^−^ transport), the ratio of active HCO_3_^−^ transport to carboxylation rate at RuBisCO is approximately 2:1 for all three temperatures. The HCO_3_^−^ concentrations within the chloroplast are ~ 20 times of that within cytosol at 10 °C, and ~ 40 times at 18 and 25 °C. This is the result of cytosolic HCO_3_^−^ concentrations remaining ~ 0.7 mM across all temperatures, whereas the chloroplast HCO_3_^−^ concentration increased from 15 mM at 10 °C to ~ 30 mM at 18 and 25 °C.

## Discussion

The CCM of *Pt* is essential to supply elevated CO_2_ concentrations around RuBisCO (Hopkinson et al. 2011; Hopkinson 2014). While there has been much focus on the response of diatom carbon fixation to ocean acidification (Wu et al. 2014; Shi et al. 2019; Wada et al. 2021), less is known about how marine heatwaves and rising sea surface temperatures (SST) may impact the diatom CCM and ultimately the ability of diatoms to sequester carbon.

### CCM activity increases with temperature

Our experimental conditions of nutrient replete, saturating light resulted in increasing carbon fixation rates in *Pt* as temperatures increased. Concomitantly, there was also an increase in CCM activity to support the increased demand of CO_2_ for fixation, overcoming the challenges at higher temperatures of lower CO_2_ solubility in the bulk medium, higher half saturation constants for CO_2_ by RuBisCO and lower CO_2_/O_2_ specificity of RuBisCO (Galmés et al. 2016). Increased activity of the CCM occurred due to an overall increase in catalytic rates of CAs and HCO_3_^−^ transporters, as well as enhanced membrane permeability to CO_2_ and increased rates of CO_2_ diffusion. As temperatures increased, we observed a shift towards a larger proportion of HCO_3_^−^ to CO_2_ uptake from the bulk media, and a slight lowering of RuBisCO saturation state (from 81 to 73%). Modeling of the CCM revealed that *Pt* initially increased CO_2_ supply to RuBisCO by enhancing its “chloroplast pump” (from 10 to 18 °C). This involved increasing HCO_3_^−^ transport across the chloroplast membranes. The rapid removal of inorganic carbon from the cytosol was quickly replaced by predominantly diffusive entry of CO_2_ but also active HCO_3_^−^ uptake into the cell. The ratio of cellular CO_2_: HCO_3_^−^ uptake did not change between 10 and 18 °C (Fig. 3c). However, between 18 and 25 °C it appeared that the “chloroplast pump” cannot be further enhanced, either unable to elevate chloroplast HCO_3_^−^ concentrations above 30 mM or unable to maintain a chloroplast-to-cytosol HCO_3_^−^ gradient greater than ~ 40 fold. Once this limit had been reached, the increased demand for inorganic carbon was supplied by enhancing the uptake of HCO_3_^−^ from the bulk media. A comparable intracellular HCO_3_^−^ concentration ~ 30 mM has been estimated in cyanobacteria previously (Woodger et al. 2005; Mangan and Brenner 2014).

### Temperature effects on kinetics or molecular regulation on CCM components?

As temperature increases, catalytic activity, diffusion rates, and membrane fluidity also increase until an optimum is reached. Observed increased rates of CA activity, HCO_3_^−^ transport, and CO_2_ carboxylation at RuBisCO are of magnitudes that could be explained by the direct impact of temperature (for enzymes assuming a Q_10_ around 2) without changes in enzyme abundance. Likewise, the differences in the magnitudes of various C_i_ fluxes (Fig. 6b, c) between 10 and 18 °C could also be entirely due to different temperature sensitivities of CCM components. However, at 25 °C the shifted ratio of CO_2_ to HCO_3_^−^ uptake fluxes (Fig. 6a) and the upheld saturation state of RuBisCO suggest that there is a temperature-driven physiological and molecular regulation of CCM components. Further investigations (e.g., transcriptional studies) beyond our current model are needed to test whether *Pt* regulates its CCM through gene expression in response to changing temperatures. Previous studies have demonstrated that CCM components (e.g., CA, HCO_3_^−^ transporters) are transcriptionally regulated via CO_2_ availability (Harada et al. 2006; Ohno et al. 2012; Nakajima et al. 2013; Hennon et al. 2015) indicating that the CCM is dynamically able to respond to changing environmental conditions.

### Temperature impacts on the energy cost of the *Pt* CCM

The energetic cost of the CCM is thought to be primarily driven by the active uptake of HCO_3_^−^ across membranes and to be strongly correlated with the amount of CO_2_ leaked from the pyrenoid. While CO_2_ leaks from the pyrenoid and chloroplast in the *Pt* CCM model, it is recycled in the cytosol by CA into HCO_3_^−^. This “chloroplast pump” model in *Pt* (Hopkinson et al. 2011; Hopkinson 2014) revealed the dependence on active pumping of HCO_3_^−^ from the cytosol into the chloroplast to maintain a low cytosolic CO_2_ concentration. This low cytosolic CO_2_ concentration drives the net influx of CO_2_ from the bulk environment into the cytosol, along with rapid recycling of CO_2_ respired from the mitochondria or leaked from the chloroplast. Below we discuss the energy costs of this model in the context of temperature but acknowledge that other diatoms have different CCM strategies such as relying on external CA for extracellular HCO_3_ uptake while CO_2_ could leak from the cell (Tsuji et al. 2017a; Li and Young 2022).

Active transport of HCO_3_^−^ across the cytoplasmic membrane in *Pt* is done by the Na^+^-dependent SLC4-type transporters while the “chloroplast pump” HCO_3_^−^ transporters are yet to be identified (Nakajima et al. 2013; Tsuji et al. 2017b; Nawaly et al. 2022). Without direct measurements, HCO_3_^−^ transport across the plasmalemma or chloroplast envelope membranes was estimated to require 0.5 or 1 ATP for each HCO_3_^−^ transported (Hopkinson et al. 2011; Raven et al. 2014). Considering the HCO_3_^−^ concentration gradient between the chloroplast and cytosol was ~ 40 fold at maximum (Fig. 6), and factoring in the ~ 20 mV chloroplast envelope membrane potential under light (Demmig and Gimmler 1983; Pottosin and Dobrovinskaya 2015), the energy input required would be ~ 11 kJ/mol HCO_3_^−^ transported from the cytosol to the chloroplast stroma, which is considerably lower than the −51 kJ/mol for the hydrolysis of ATP (estimated from chloroplast ATP/ADP concentrations (Heineke et al. 1991)). This indicates that a relatively energy efficient “chloroplast pump” is possible, so the estimate of 0.5 ATP for each HCO_3_^−^ transported can hold. In contrast to HCO_3_^−^ transport into the chloroplast, the cytoplasmic uptake of HCO_3_^−^ has a favorable concentration gradient, i.e., the cytosolic HCO_3_^−^ concentration is lower than the bulk environment. This favorable concentration gradient, together with the unfavorable cytoplasmic membrane potential −84 mV (resting potential, oscillating up to −40 mV) measured from the diatom *Odontella sinensis* (Taylor 2009), points to an estimated energy requirement of ~ 5.5 kJ/mol for HCO_3_^−^ transport across the cytoplasmic membrane into the cytosol. Thus, HCO_3_^−^ transporters (such as the SLC4-type) on the cytoplasmic membrane can operate efficiently using 0.5 or less ATP per HCO_3_^−^ uptake into the cytosol. The ratio of total active HCO_3_^−^ transport to CO_2_ fixed at RuBisCO remained at 2:1 across all temperatures (Fig. 6) indicating that the energy cost of the *Pt* CCM per CO_2_ fixed does not change with temperature unless the energetic costs for chloroplast and cytoplasmic HCO_3_^−^ uptake are different. Nevertheless, higher photosynthetic rates at higher temperatures (under nutrient replete conditions) will need a higher total energy input, requiring higher saturating light intensities.

In addition to the energetic cost of HCO_3_^−^ transport, energy costs and resource allocation of the CCM must also take into consideration the synthesis and maintenance of the CCM biophysical components (Raven et al. 2014). To compensate for slow catalytic rates at cold temperatures, many CCM components and RuBisCO are likely increased in abundance (Young et al. 2015; Li and Young 2022), this would require a larger investment in resources, such as nitrogen. While the abundance of other CCM components were not directly measured in this study, RuBisCO abundance did not change with temperature.

### Combined effects of increasing SST and ocean acidification

Climate change will lead to both increasing SST and lowering of ocean pH. While warming SST leads to lower solubility of CO_2_ in seawater with a given pH, increasing atmospheric CO_2_ leads to elevating dissolved CO_2_ and HCO_3_^−^ in the ocean, decreasing the oceanic pH (Zeebe 2012; Cheng et al. 2022). We hypothesized that the effect of rising temperatures would put additional pressure on the CCM, negating any relaxation of the CCM due to increasing CO_2_ concentrations. Our study showed that *Pt* increases CCM activity with increasing temperature, maintaining RuBisCO saturation above 70%. However, the increase in CCM activity is in pace with faster carboxylation rates, so that the estimated cost of the CCM does not increase per carbon fixed. Our study was conducted under nutrient replete conditions over a temperature range that led to increased carbon fixation rates with temperature. Nutrient limitation is predicted to become more prevalent in conjunction with warming SST due to enhanced stratification and could counter any temperature-driven increases in growth rate (Marinov et al. 2010). How the CCM functions under higher temperatures when nutrient limitation restricts carbon fixation rates has yet to be elucidated though there is evidence of either the Calvin cycle or CCM being downregulated in phytoplankton under phosphate limitation (Beardall et al. 2005; Brembu et al. 2017). Our study also highlighted the dependence of HCO_3_^−^ transport in *Pt*’s “chloroplast pump” and an upper limit of either chloroplast HCO_3_^−^ concentrations or chloroplast-to-cytosol gradients. At this limit (above 18 °C in this study), the CCM is no longer sensitive to CO_2_ availability and ocean acidification would not affect carbon fixation rates under these experimental conditions.

## Supplementary Information

Below is the link to the electronic supplementary material.

Supplementary file1 (PDF 1027 KB)

## Data Availability

The Python script for Pt CCM modeling is available on GitHub: https://github.com/limengwsu/Pt_CCM_T. The results summary table is available on Dryad: 10.5061/dryad.jdfn2z3fn and raw MIMS signal data files are available upon request to the corresponding author.
