# Supplementary material for: Temperature sensitivity of carbon concentrating mechanisms in the diatom Phaeodactylum tricornutum
Source: Photosynth Res. 2023 Mar 7;156(2):205–15. doi: 10.1007/s11120-023-01004-2 (PMC10154264; doi:10.1007/s11120-023-01004-2)
Supplement: Supplementary file 1 — Supplementary file1 (PDF 1027 KB) [file 11120_2023_1004_MOESM1_ESM.pdf]

Supplementary Information for

Temperature Sensitivity of Carbon Concentrating Mechanisms in the Diatom *Phaeodactylum tricornutum*

Meng Li[1], Jodi N. Young[1]

[1]School of Oceanography, University of Washington, Seattle, WA.

ORCIDs:

Meng Li: 0000-0001-9831-8575

Jodi N. Young: 0000-0002-3156-8403

Corresponding author:

Jodi N. Young, Email: youngjn@uw.edu

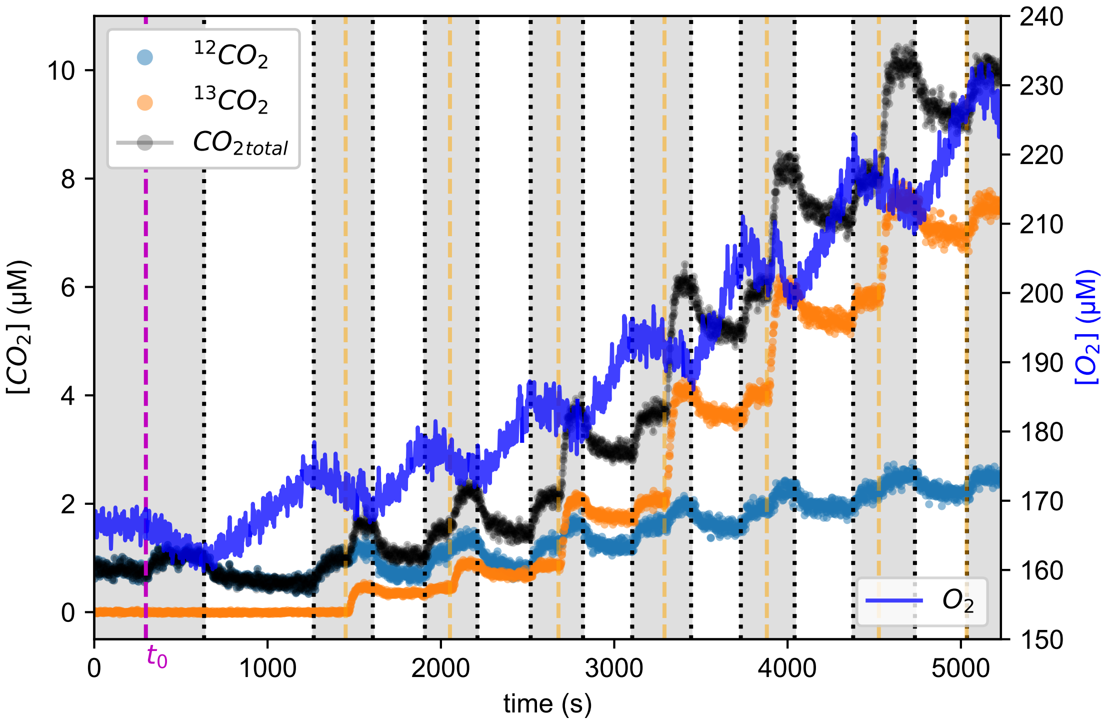

Fig. S1 Signal traces of an experiment at 25°C with sequentially increasing DIC. Shaded areas denote dark periods (light off) for MIMS chamber and *Pt* Cells. Dotted lines mark light on and light off events. Orange dashed lines mark events of adding ^{13}C-$NaHCO_3$ while t_0 marks the time point of adding *Pt* cells.

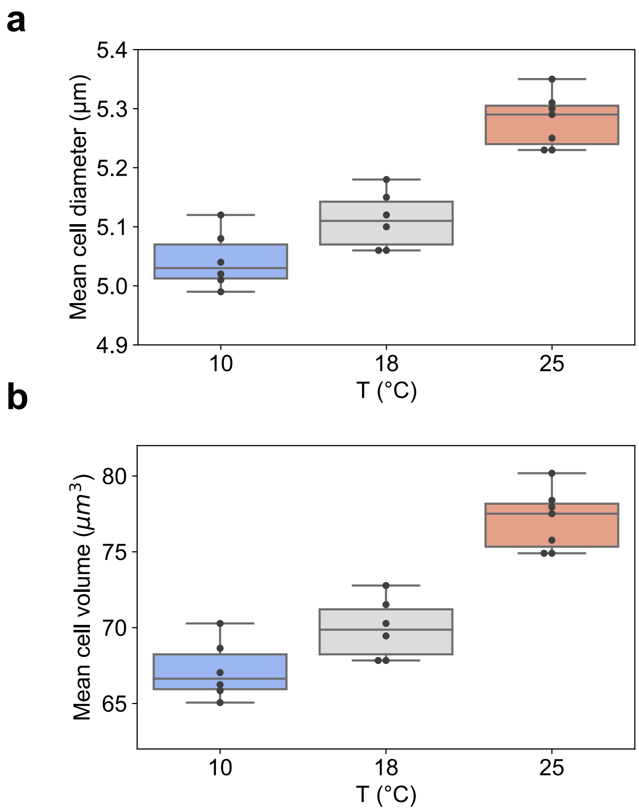

Fig. S2 *Pt* cell size partially explains leveling-off growth rate at higher temperature. Average cell diameter **(a)** and volume **(b)** observed for *Pt* cultured under different temperatures.

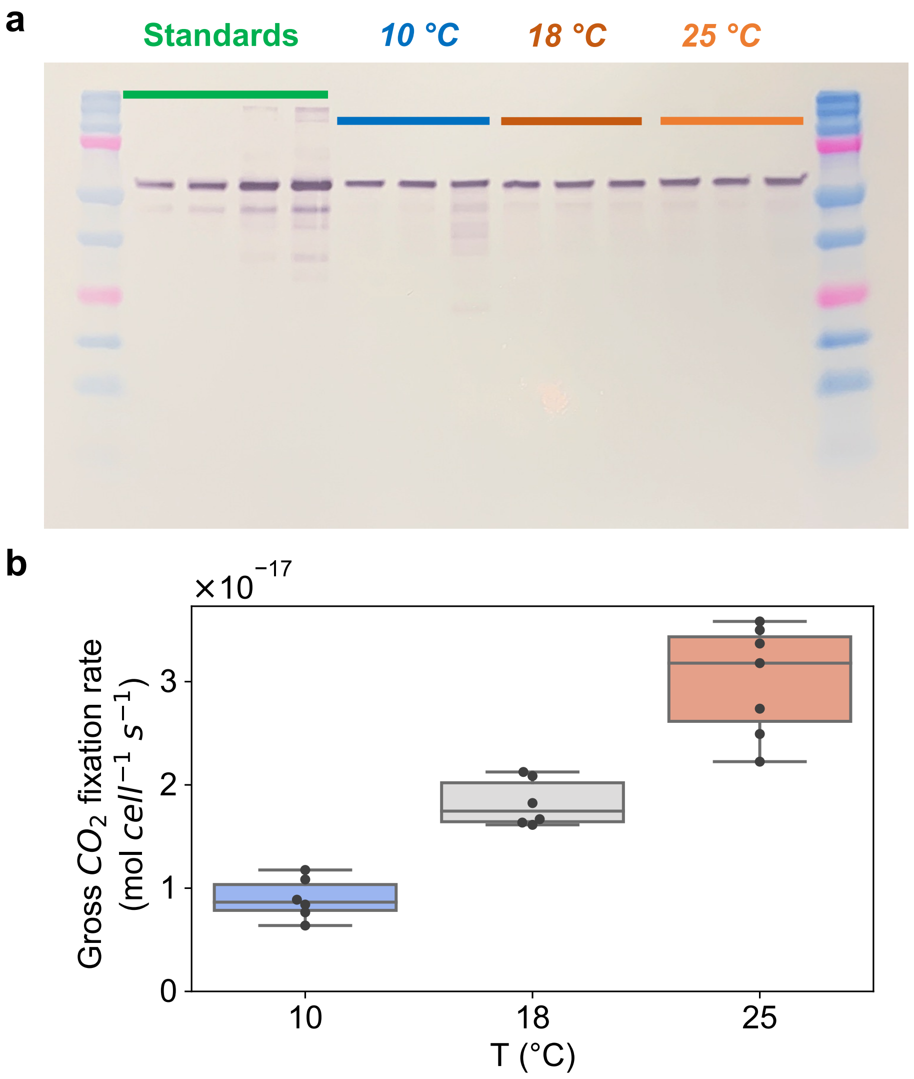

Fig. S3 Temperature impact on RuBisCO quantity and gross CO_2 fixation rate. **a.** Western blot quantification of RbcL using Agrisera standard and antibody. **b.** Box plot of gross CO_2 fixation rate at RuBisCO by adding respiration rate to net O_2 evolution rate at different temperatures.

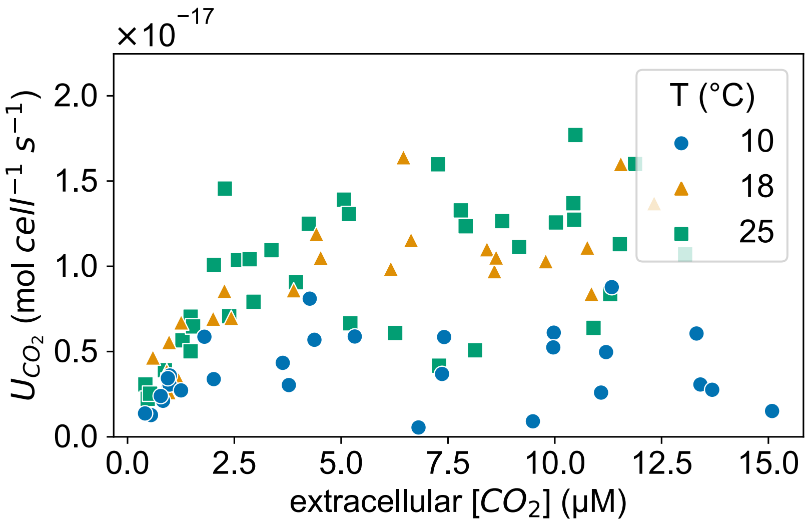

Fig. S4 CO_2 uptake (U_{CO2}) versus extracellular CO_2 concentration. Data points were pooled from 4, 3, 5 independent cultures for temperature at 10, 18, 25 °C respectively.

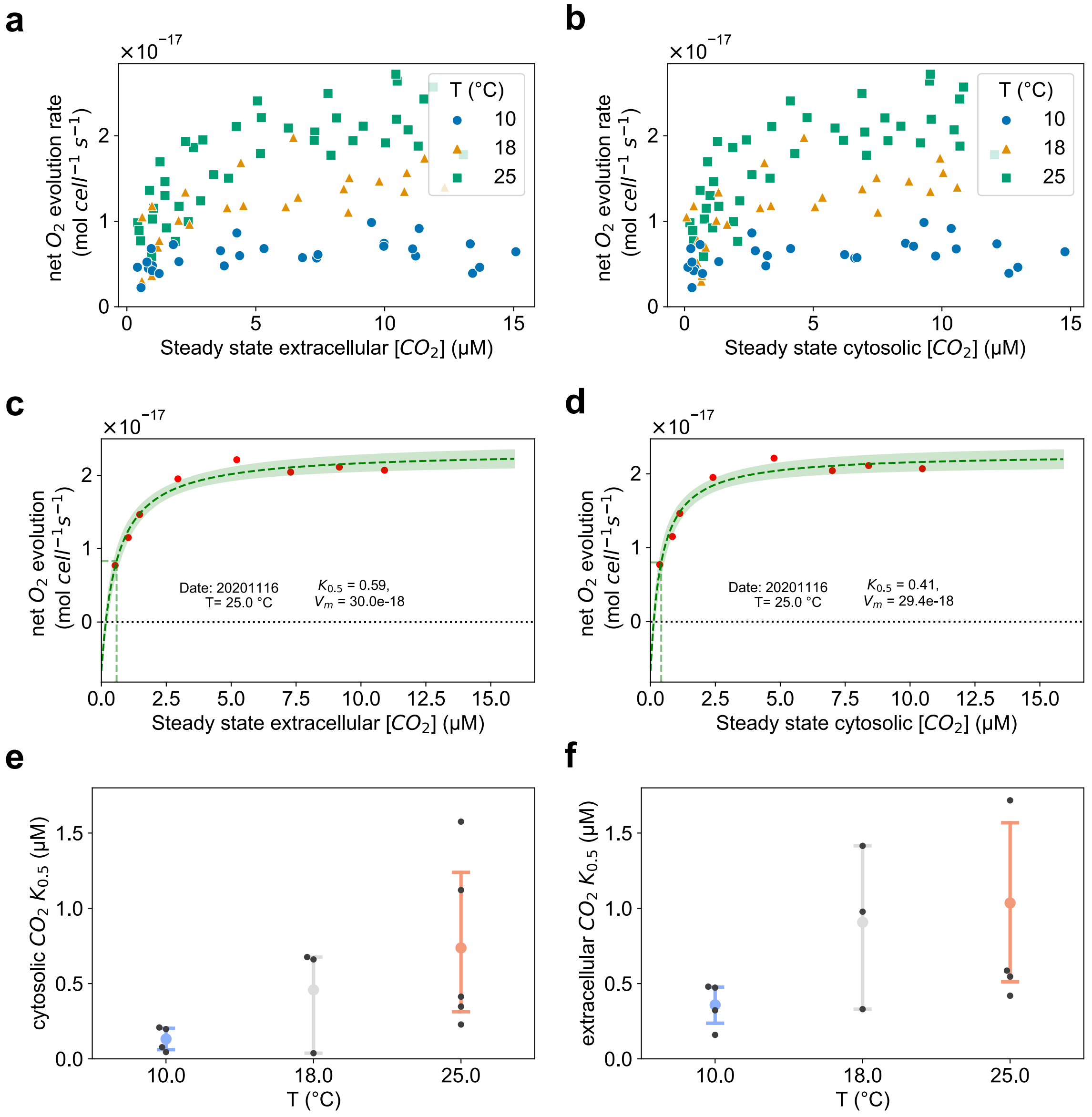

Fig. S5 Net O_2 evolution rate in *Pt* as a function of extracellular and cytosolic CO_2 concentration. **a** and **b** Pooled data points of correlation between net photosynthetic rate and CO_2 concentration outside (a) and inside (b) the cell. Cytosolic CO_2 concentration is estimated from extracellular CO_2 concentration from **Eq 1** (see Materials and Methods). **c** and **d.** Examples of fitting net O_2 evolution rate as a function CO_2 concentration to Michaelis-Menten equation. Green dashed curve and shade show best fit and 95% confidence interval. Text insert shows the experimental date, temperature and best fit parameters, with Vm representing the gross carboxylation rate factoring in respiration. **e** and **f.** The distribution and statistics of Michaelis constant ($K_{0.5}$) of CO_2. Error bars denote 95% of confidence interval. Each data point is from an experiment with an independent batch culture at different temperature.
